# The Evolution of an Intensive Support Team in an Intellectual Disability Service - a 10 year review.

**DOI:** 10.1192/j.eurpsy.2026.10434

**Published:** 2026-07-31

**Authors:** E. V. Crossey, B. MacPherson

**Affiliations:** Intellectual Disability, NHS Lothian, Edinburgh, United Kingdom

## Abstract

**Introduction:**

The Mental Health Intensive Support and Treatment Team (MHIST) developed as a part time, 9am-5pm weekday team in 2008 due to increasing numbers of patients with Learning Disability (LD) being regularly admitted to General Adult Psychiatry (GAP) beds (10 patients In Nov 2008), the concerns of GAP staff that they hadn’t the knowledge, skills, confidence or experience to effectively serve the needs of this client group, and the likelihood that without service reprovision, in a climate of ongoing bed reductions and higher incidences of mental health problems in the LD population, LD patients with comorbid psychiatric presentations would not be equally or effectively treated.

Over time, the service replaced the CLDT input to GAP wards, and the team became established with increased nursing, psychiatry and dedicated AHP input. The remit also expanded to include assertive outreach / crisis intervention to focus on prevention of admissions, and to support discharge and stabilisation on return home through extending intensive nursing and Psychiatry input. Again following review the service expanded to cover 7 days per week. We were also able to provide MHAS assessments from 2018 as a means of further preventing admissions and promoting home treatment options, but also to provide a more specialist service to those at MHAS in crisis.

**Objectives:**

To review the MHIST service over the past ten years to assess what the Gold Standard of the MHIST Team and other Intensive Support Teams would look like.

**Methods:**

Review of the patient database over the past 10 years and collating and reviewing the data to help us better understand our patient groups needs, their numbers and demographics, how this has changed pre and post Covid 19 pandemic, and how this might project into the coming years. Surveys were also carried out with the CLDTs, MHAS team and other associated services that utilised our service for their current views on the team and what they feel would be beneficial in the future. We also reviewed feedback from both patients, carers and families on their views of the MHISt team and impact of our input.

**Results:**

The feedback from GAP inpatient staff, MHAS staff and the CLDTs were mainly positive and seeking more input from MHIST in times of crisis.

Patient and carer feedback was also hugely positive and has shown the much needed input from the team.

**Conclusions:**

Now following a service review we look ahead at what the future of the MHIST team holds and what the Gold Standard of an Intensive Support Team would involve.

**Disclosure of Interest:**

None Declared

